# Efficacy of chemical shift MRI for differentiating diffuse red bone marrow reconversion and hematological malignancies

**DOI:** 10.3906/sag-1812-125

**Published:** 2019-04-18

**Authors:** Burcu AKMAN, Hatice Ayça ATA KORKMAZ, Ahmet SARI

**Affiliations:** 1 Department of Radiology, Kanuni Research and Education Hospital, Trabzon Turkey; 2 Department of Radiology, Faculty of Medicine, Farabi Hospital, Karadeniz Technical University, Trabzon Turkey

**Keywords:** Magnetic resonance imaging, in-phase, out-of-phase, chemical shift, reconversion, hematological malignancy, red bone marrow

## Abstract

**Background/aim:**

The main purpose of our study was to determine the efficacy of chemical shift imaging (CSI) for differentiating diffuse red bone marrow reconversion (RBMR) and hematological malignancies. We also aimed to calculate the cut-off value for these entities with similar imaging features in routine magnetic resonance (MR) sequences.

**Materials and methods:**

A total of 54 patients were included: 17 patients (31.4%) with hematological malignancies (group 1), 16 patients (29.6%) with RBMR (group 2), and 21 patients (38.0%) with no clinical and hematological malignancies (control group). Patients with no pathological data or completed two-year follow-up and children were excluded from the study. An experienced radiologist on MRI evaluated the images blindly for final diagnosis. Pathologic results were determined as gold standard. Regions of interests (ROI) were placed on the vertebrae in CSI and signal intensity ratios (SIR) were calculated. The cut-off value was calculated using receiver operating characteristic (ROC) analysis.

**Results:**

SIR values were 0.97 ± 0.16, 0.69 ± 0.31 and 0.28 ± 0.35 (P < 0.001) for GI, G2, and G3, respectively. The cut-off value was 0.82 (P < 0.001). The sensitivity rate was 83.3% (AUC: 58%–96%), specificity was 87% (AUC: 58–98).

**Conclusion:**

CSI may be a valuable diagnostic tool for differentiating diffuse RBMR and hematological malignancies.

## 1. Introduction

Normal bone marrow contains water, fat, and cellular elements. The red bone marrow (RBM) contains 40% of water (free and bound), 20% of protein, and 40% (25%–50%) of fat. In contrast, the yellow bone marrow (YBM) contains 15% of water, 5% of protein, and 80% of fat (1–4). In newborns, the bone marrow consists mainly of active RBM. After the infantile period, the RBM begins to transform into YBM. When the active bone marrow is not able to meet the increased hematopoiesis required by the body, YBM converts to RBM, and this process is called reconversion (3–7). The causes of reconversion can be classified under medical and nonmedical conditions. Medical conditions include obesity, respiratory disorders, diabetes, anemias, patients receiving granulocyte colony stimulating factor therapy, chemotherapy, and radiotherapy. Nonmedical conditions include heavy smoking and practicing sports requiring high oxygen such as marathon running or freediving (2,5–7).

Magnetic resonance imaging (MRI) is an ideal noninvasive imaging modality for evaluating bone marrow (1,4). Routine spinal MR protocols include T1 weighted (T1W), T2 weighted (T2W), and short tau inversion recovery (STIR) sequences (3). Due to its high water content, RBM has an intermediate low signal intensity in T1W and low signal intensity in T2W. Due to its high-fat content YBM has high signal intensity in T1W and T2W (2,4,6).

In T1W, abnormal bone marrow and RBM have a diffusely equal or lower intensity than the adjacent muscle or disc. In RBM reconversion and malignancies, we can see the high signal intensity in STIR and T2 weighted fat suppressed images (2,6). Therefore, it is near impossible to distinguish hyperplastic red marrow from other bone marrow replacement disorders while observing only conventional MRI techniques (1). Due to its similar imaging features, leukemia, lymphoma, and breast cancer metastases with widespread bone involvement are difficult to distinguish from RBMR (3).

Chemical shift MRI is commonly used for liver and adrenal glands imaging for detecting lipid content and characterizing lesions (8–11). Also, chemical shift MRI is the best sequence for differentiating malignant and benign bone marrow lesions (3). Chemical shift MRI is based on the different oscillation frequency of water and fat protons (8,10). Due to the presence of fat and water in the normal bone marrow, bone marrow signal intensity is suppressed in the out-of-phase. In malignant infiltration of the bone marrow, tumoral structures are replaced by fat components of the bone marrow and no regression occurs (10–12).

 The main purpose of our study was to determine the efficacy of chemical shift imaging (CSI) in the differential diagnosis of red bone marrow reconversion (RBMR) and diffuse bone marrow involvement in hematological malignancies. We also aimed to calculate the cut-off value for the differential diagnosis of RBMR and hematological malignancies.

## 2. Materials and methods

### 2.1. Study population

After ethical committee approval (Karadeniz Technical University Faculty of Medicine Assessment of the Scientific Research Committee Approval Number: 2010/7), we recruiteda total of 54 patients (32 female and 22 male), 17 patients (31.4%) with hematological malignancies (group 1), 16 patients (29.6%) with RBMR (group 2), and 21 patients (38.0%) with no clinical and hematological malignancies (control group).

The vertebral bone marrow signal intensity of the hematological malignancy cases (group 1) and cases with RBM reconversion (group 2) was diffusely equal to or lower than those of the neighboring muscle or disc in T1-weighted images. In the control group (group 3), vertebral bone marrow signal intensity in T1-weighted images was higher than those of the neighboring muscle or discs. Patients with no pathological data, completed two-year follow-up, and children were excluded from the study. Pathological diagnosis was accepted as the gold standard method.

### 2.2. MR technique

Siemens Magnetom (Symphony and Aera; Siemens Healthcare, Erlangen, Germany) 1.5T MRI systems and a dedicated standard spine coil were used in this study.

The protocols used were sagittal T1W turbo spin-echo (TSE) (TR / TE: 343/14) and axial–sagittal T2-W TSE (TR / TE: 4000/106) sequences. No contrast agent was administered during the examination.

The sequences used in the selected cases were: T1W images, matrix of 291 × 448 mm, “number of acquisition” (NEX) 1 and field of view (FOV) for the cervical region was 250 × 384 mm, for the thoracic region was 300 × 300 mm, and for the lumbar regions was 300 × 300 mm.

Cross-sectional thickness was 4 mm and the range was 0.4 mm. On T2W images, matrix chosen was 192 × 384 mm, NEX = 2, FOV = 250 × 384 mm for cervical, 300 × 300 mm for thoracic, and 300 × 300 mm for lumbar regions with cross-sectional thickness of 4 mm and a cross-section of 0.4 mm. Conventional MRI in sagittal plane was obtained with T1W TSE (TR / TE: 343/14) and T2W axial and sagittal plane with TSE (TR / TE: 4000/106) sequences.

Chemical shift imaging protocol: In and out-of-phase MR images were obtained in sagittal projection (in-phase TR/TE: 118/5.27, out-of-phase TR/TE: 118/2.35). In CSI, the matrix selected was 270 × 512 mm, NEX = 1 and FOV = 262 × 350 mm, the cross-sectional thickness was 4 mm, and the cross-sectional range was 0.4 mm. No contrastagents were applied during the examination. Vertebral bone marrow chemical shift MR imaging was completed in approximately 40–50 s.

### 2.3. MR image analysis

A neuroradiologist with 10 years of experience evaluated the thoracolumbar MRI’s of all the groups. The radiologist was blinded to the final diagnosis and clinical history. In all the groups the T1W and CSI images of each patient were evaluated in one special workstation (Leonardo, Siemens).

**Figure 1:**
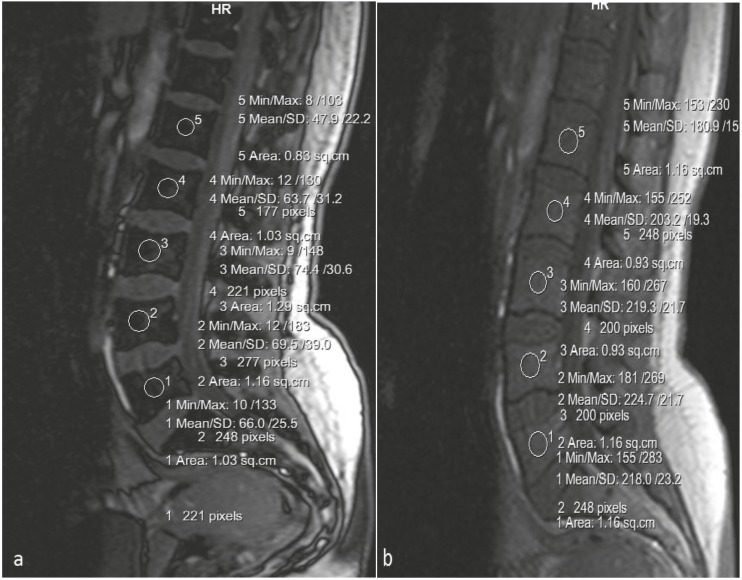
SI measurements on out-of-phase and in-phase sequences.

At first, ROIs were placed on the vertebrae bodies’ midsections in both sequences of CSI in at least five vertebrae and then SI measurements were analyzed (Figure 1). The mean area of circular ROIs was determined as 0.8–1.1 cm2. Mean SI values of the vertebrae in out-of-phase and in-phase sequences were calculated. Signal intensity ratio (SIR) was calculated with the obtained values according to the “SIR = out-of-phase signal intensity value / in-phase signal intensity value” formula which was used in previous studies (12–14) to distinguish benign from malignant bone marrow involvement. The cut-off value for discriminating malignancies was calculated using ROC analysis.

### 2.4. Statistical analysis

In this study, the Chi-square test was used for counting data. The Kolmogorov–Smirnov test was used to evaluate the values ​​obtained by the ROI measurements to determine if they have a normal distribution or not. Student-t-test was used to evaluate the two parameters that matched normal distribution, and ANOVA test was used to evaluate the remaining parameters. Mann–Whitney U test was used for two nonparametric tests, and Kruskal–Wallis was used for more than two parameters. Pearson (parametric) and Spearman (nonparametric) analysis were used for correlation analysis.  P < 0.05 values were considered statistically significant. All analyzes were done using the SPSS 22.0 statistical program (IBM Corp., Armonk, NY, USA) (15).

## 3. Results

Demographic characteristics, hemoglobin (Hb) levels, clinical diagnoses, BM biopsy results, and SIR values were shown in Table 1–4.

**Table 1:** Demographic characterictics, hemoglobin levels, clinical diagnoses, SIR values, and biopsy results of group1.

Case	Sex	Age	Weight	Hb level(mg/dl)	History of hematological malignancy	SIR	**BM biopsyresults
1	Male	61	73	7	*(+)	1.1	CLL+ NHL
2	Male	68	78	10	*(+)	1.32	B cell lymphoma
3	Male	68	80	9	*(+)	0.72	MM
4	Female	38	85	12	*(+)	0.99	ALL
5	Female	62	86	8	*(+)	0.99	CLL
6	Male	48	79	9	*(+)	0.8	MM
7	Male	74	64	8	*(+)	0.86	MM
8	Male	68	71	7	*(+)	0.83	MM
9	Male	45	68	8	*(+)	1.06	MM
10	Female	44	95	11	*(+)	1.23	MM
11	Female	70	70	7	*(+)	0.85	MM
12	Female	55	80	12	*(+)	0.98	MM
13	Female	51	80	9	*(+)	0.95	B cell lymphoma
14	Male	77	70	10	*(+)	0.83	MM
15	Male	52	72	7	*(+)	1.02	MM
16	Female	21	50	9	*(+)	1.2	HL
17	Female	42	51	8	*(+)	0.86	AML

Hb: Hemoglobin, SIR: Signal intensity ratio, BM: Bone marrow, CLL: Chronic lymphocytic leukemia, NHL: Non-Hodgkin’s Lymphoma, MM: Multiple myeloma. ALL: Acute lymphoblastic leukemia. *(+): Patient with history of hematological malignancy. **: Bone marrow results at at the time of MRI imaging.

**Table 2:** Demographic characterictics, hemoglobin levels, clinical diagnoses, SIR values, and biopsy results of group 2.

Case	Sex	Age	Weight	Hb level (mg/dl)	History of hematological malignancy	SIR	**BM biopsy results
1	Female	23	54	13	***( - )	0.58	No BM biopsy Stable at follow-up
2	Female	41	69	12	***( - )	0.8	No BM biopsy Stable at follow-up
3	Female	27	70	11	***( - )	0.67	No BM biopsy Stable at follow-up
4	Male	46	85	10	***( - )	0.5	Hypercellular BM
5	Female	37	65	13	***( - )	0.81	Normocellular BM
6	Male	61	70	10	***( - )	0.73	Hypercellular BM
7	Female	25	90	14	***( - )	0.59	No BM biopsy Stable at follow-up
8	Male	61	75	10	***( - )	0.65	No BM biopsy Stable at follow-up
9	Male	35	60	9	***( - )	1.22	Hypercellular BM
10	Female	39	88	7	*(+)	0.63	Hypercellular BM
11	Male	34	70	7	*(+)	1.3	Hypercellular BM
12	Male	46	74	9	*(+)	1.16	Hypercellular BM
13	Female	54	78	13	***( - )	0.69	No BM biopsy Stable at follow-up
14	Female	33	72	13	***( - )	0.27	No BM biopsy Stable at follow-up
15	Male	41	89	14	***( - )	0.21	No BM biopsy Stable at follow-up
16	Female	51	60	11	***( - )	0.35	No BM biopsy Stable at follow-up

Hb: Hemoglobin, SIR: Signal intensity ratio, BM: Bone marrow *( + ): Patients who had history of hematological malignancy. **: BM results at at the time of MRI imaging. ***( - ): Patient who had no history of hematological malignancy.

**Table 3:** Demographic characteristics, hemoglobin levels, clinical diagnoses, and SIR values of group 3.

Case	Sex	Age	Weight	Hb level(mg/dL)	History of hematological malignancy	SIR
1	Female	51	51	11	***( - )	0.2
2	Female	45	70	13	***( - )	0.3
3	Female	43	70	13	***( - )	0.3
4	Male	35	68	13	***( - )	0.29
5	Female	45	60	15	***( - )	0.48
6	Female	40	75	12	***( - )	0.26
7	Female	40	51	13	***( - )	0.31
8	Female	29	49	12	***( - )	0.21
9	Male	45	60	14	***( - )	0.21
10	Male	33	80	14	***( - )	0.24
11	Female	48	120	9	***( - )	0.39
12	Female	49	70	14	***( - )	0.41
13	Male	45	71	14	***( - )	0.24
14	Male	31	76	14	***( - )	0.21
15	Female	42	68	15	***( - )	0.21
16	Female	50	68	14	***( - )	0.27
17	Female	56	60	13	***( - )	0.26
18	Female	33	60	13	***( - )	0.2
19	Female	51	83	12	***( - )	0.22
20	Male	58	71	13	***( - )	0.4
21	Female	56	90	13	***( - )	0.37

Hb: Hemoglobin, SIR: Signal intensity ratio ***( - ): Patient who had no history of hematological malignancy.

**Table 4:** Mean SIR values for each group

Groups	Mean SIR values	SD
Group 1	0.97	±0.16
Group 2	0.69	±0.31
Group 3	0.28	±0.35

SIR: Signal intensity ratio, SD: Standard deviation

**Figure 2:**
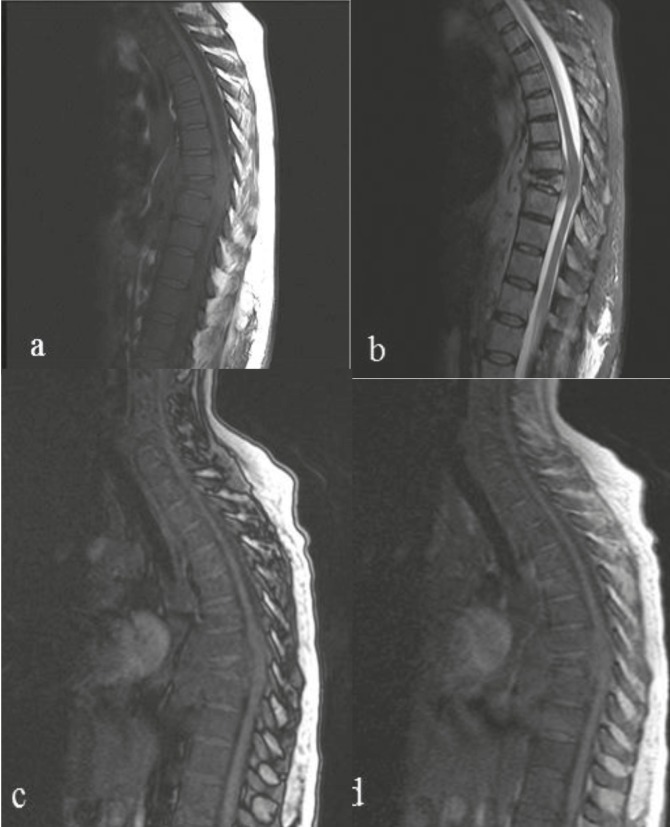
A 51-year-old female patient, biopsy result of T8 vertebra consisting of B-cell lymphoma. (a) Thoracic vertebral bone marrow signal intensity was low in the sagittal T1W sequence compared to the adjacent disc. T8 vertebra had pathological fracture and epidural mass was shown at the same level. (b) The pathological fracture of the T8 vertebra and the epidural mass in the neighborhood were more prominent in the thoracic sagittal T2W sequence. In sagittal out-of-phase sequence (c) no signal loss was observed compared to the in-phase sequence (d) SIR was calculated as 0.95 on chemical shift sequances and higher than the cut off value.

In group 1 (hematological malignancies with diffuse bone marrow involvement) the vertebral bone marrow signal intensity was diffusely equal to or lower than the adjacent muscle or disc in T1-weighted images. In CSI, no signal loss was observed in the out- of-phase sequence compared to the in-phase sequence (Figure 2). The mean OP / IP SIRs of the cases were calculated as 0.97 ± 0.16. All the patients in group 1 had a result of pathologic bone marrow biopsy at the time of MRI imaging that revealed infiltration in accordance with the hematological malignancy.

**Figure 3:**
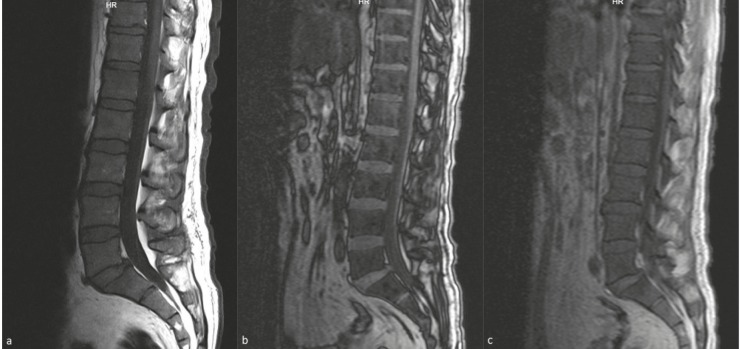
A 60-year-old male patient had chronic disease anemia. Bone marrow biopsy was consistent with hypercellular bone marrow. (a) The vertebral bone marrow signal intensity in the lumbar sagittal T1W sequence is equal to those in T1W images compared to the adjacent disc. In the sagittal out-of-phase sequence (b), there was signal loss compared to the in-phase sequence (c). The SIR value is calculated as 0.73 and is consistent with benignity.

In group 2 (cases with RBM reconversion) vertebral bone marrow signal intensity was diffusely equally or lower than the adjacent muscle or disc in T1-weighted images. In CSI of the 3 patients (18.25%), the signal loss was not observed in the out-of-phase sequence while in the remaining 13 (81.25%) patient’s images, the signal loss was observed in the out-of- phase sequence compared to the in-phase sequence (Figure 3). In CSI, the mean OP / IP SIRs of the cases were calculated as 0.69 ± 0.31. Three patients in this group had a history of hematological malignancy; one patient had a history of T-cell lymphoma, one had an acute myelocytic leukemia (AML), and one had an acute lymphoblastic leukemia (ALL). In these three cases, bone marrow biopsy at the time of MRI showed no infiltration, and hypercellular bone marrow was detected.

**Figure 4:**
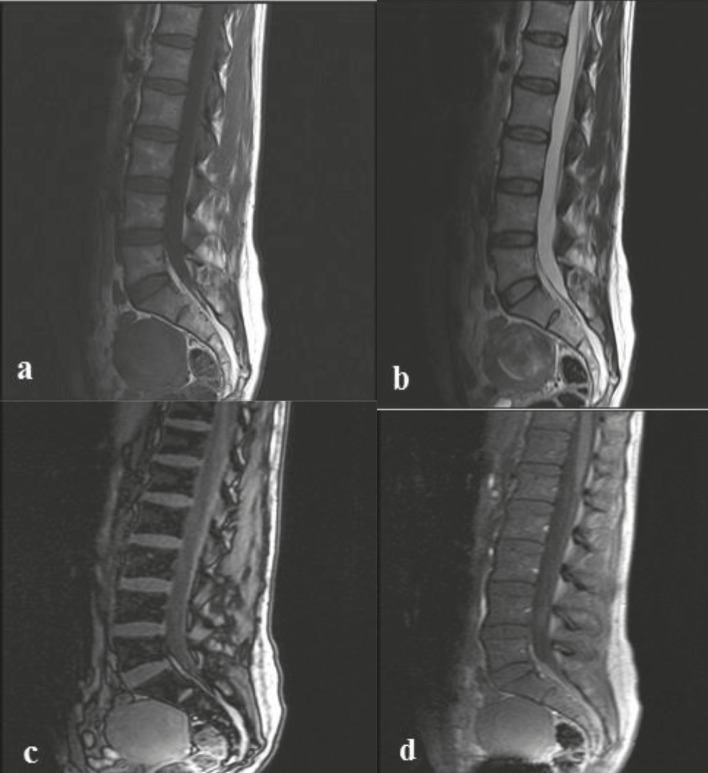
A 45-year-old woman with no history of chronic disease or malignancy with a normal hemoglobin value (control group). (a) In the sagittal T1W sequence of the lumbar vertebrae, the bone marrow signal intensity was higher than the adjacent disc and was normal. (b) In T2W sequence, vertebral bone marrow was homogeneous and no pathology was detected. In the sagittal out-of-phase sequence (c), there was marked loss of signal in the vertebral bone marrow compared to the in-phase sequence (d). The SIR value was calculated as 0.3 and was lower than the cut-off value. The patient was stable during clinical and radiological 2-year follow-up and no malignancy developed.

In group 3 (control group), the vertebral bone marrow signal intensity in T1-weighted images was higher than the adjacent muscle or disc. CSI in all cases showed a significant loss of signal in the out-of-phase sequence compared to the in-phase sequence (Figure 4). The mean OP / IP SI ratios of the patients were calculated as 0.28 ± 0.35. 

In our study, according to ROC analysis, AUC was calculated to be 0.828 (95%), CI (safety interval) was 0.647–0. 989, P = 0.002. ROC analysis revealed a cut-off value of 0.82 for the differentiation of hematological malignancies from RBMR. Those with lower SIR values than the cut-off value were considered benign bone marrow, and those with higher values than the cut-off value were considered malignant bone marrow.

The cut-off value for hematological malignancy was 0.82; of the 17 patients with malignancy in group 1, 15 (88.2%) patients had higher SIR values than 0.82, of the 16 patient with RBMR in group 2, 13 (81.3%) patients were found to have lower SIR values than 0.82. Two patients with low cut-off values in group 1 were diagnosed with multiple myeloma (MM) and these were accepted as false negative results. Three patients with high cut-off values in group 2 were determined as false positive results. Two of them were patients with hematological malignancies in remission (T-cell lymphoma and AML) and with hypercellular bone marrow biopsy results. One of the false positive patients had a transverse myelitis disease and no history of malignancies.

In group 2, the patient with ALL in remission, the SIR value was calculated as 0.63. This SIR value was lower than 0.82 and was consistent with the pathologic result.

For the cut-off value of 0.82, the sensitivity of our study was calculated as 83.3% (AUC: 58%–96%), the specificity of our study was calculated as 87% (AUC: 58–98), the negative predictive value of our study was calculated as 81% (AUC: 54%–95%), and finally the positive predictive value calculated was 88% (AUC: 62%–98%).

**Figure 5:**
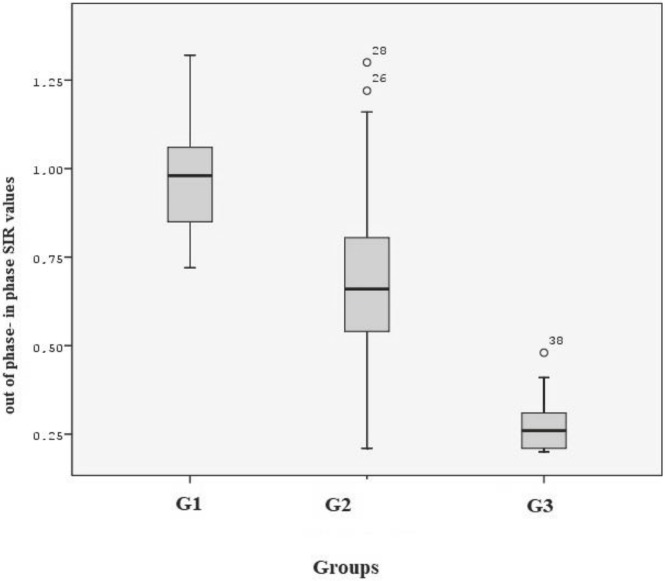
Graphical view of the distribution of SIR in all groups (Box plot).

When the cut-off value was determined as 0.82, there was a statistically significant difference between the hematological malignancies and RBMR (P < 0.001) (Figure 5).

## 4. Discussion

BMR is a confusing entity in radiology due to its similar imaging features with hematological malignancies. Various diagnostic methods such as MRI, scintigraphy, PET, and biopsy are used for differential diagnosis in hematology. However, scintigraphy and PET tests are not available in every health center and havehigh radiationdoses.

CSI is a simple, noninvasive, and fast radiological imaging technique that can be applied in all MRI systems. The most important advantage is that it can detect a small amount of fat in a mixture of water and fat (16). In CSI, bone marrow signal intensity is suppressed in the out-of-phase due to the presence of both fat and water in healthy individuals. Secondary to malignant infiltration in the bone marrow, fatty components are replaced by tumoral structures and the expected suppression does not occur (10). 

The major disadvantages of CSI include the inability to suppress signals from adipose tissue in the out-of-phase images and the difficulty of detecting small tumors embedded in fat. In addition, signal intensities from peripheral fat tissues may invalidate signal intensities of the tumor (16).

The first study on the use of CSI in bone marrow lesions was performed by Wismer GL et al. (17). To the best of our knowledge, there are no other studies focusing on the use of CSI for the differentiation of RBMR and diffuse bone marrow involvement in hematological malignancies. However, there are studies investigating the contribution of CSI in the differentiation of benign-malignant fractures in the bones (10,14,18) and in the differential diagnosis of focal bone lesions (12,13,19,20). In these studies, SIR values of neoplastic compression fractures and focal malignant lesions were found to be significantly higher than the SIR values of benign fractures and lesions.

In the present study, the malignant bone marrow SIR values were also significantly higher than the benign bone marrow SIR values. In previous studies, only focal bone marrow lesions were evaluated for the diagnostic value of CSI for differential diagnosis between malignant and benign lesions. In our study, we investigated the effıcacy of CSI for the differential diagnosis of diffuse RBM reconversion and hematological malignancies with diffuse bone marrow involvement. Early et al. determined the cut-off value for differentiation of benign and malignant bone fractures as 0.80, and Disler et al. determined the cut-off value for differentiation focal bone lesions as 0.81 (13,18). According to our study results, the cut-off value was 0.82 for the differential diagnosis of hematological malignancies and RBMR. Lower SIR values determined benign bone marrow, and those with higher values were considered malignant bone marrow. In our two false negative MM cases in group 1, the result may be secondary to diffuse but heterogeneous vertebral bone marrow involvement.

The most important limitation of our study was the small sample size. Our second major limitation is the fact that all of the biopsies were performed from the iliac crests and not directly from the vertebrae except in one patient. Finally, the present study was a retrospective study; therefore, the SIRs values were not considered in the clinical management. 

According to our study results, chemical shift MRI may be a valuable diagnostic tool for the differential diagnosis of hematological malignancies and benign disorders with RBM involvement when appropriate cut-off values are used. Further studies with larger series are required to enrich the findings of the present study.

## Acknowledgment

This research was presented as an oral presentation in the 39th National Radiology Congress, Antalya, Turkey, 6–11 November 2018.
